# Lifelong Reduction of LDL-Cholesterol Related to a Common Variant in the LDL-Receptor Gene Decreases the Risk of Coronary Artery Disease—A Mendelian Randomisation Study

**DOI:** 10.1371/journal.pone.0002986

**Published:** 2008-08-20

**Authors:** Patrick Linsel-Nitschke, Anika Götz, Jeanette Erdmann, Ingrid Braenne, Peter Braund, Christian Hengstenberg, Klaus Stark, Marcus Fischer, Stefan Schreiber, Nour Eddine El Mokhtari, Arne Schaefer, Jürgen Schrezenmeier, Diana Rubin, Anke Hinney, Thomas Reinehr, Christian Roth, Jan Ortlepp, Peter Hanrath, Alistair S. Hall, Massimo Mangino, Wolfgang Lieb, Claudia Lamina, Iris M. Heid, Angela Doering, Christian Gieger, Annette Peters, Thomas Meitinger, H.-Erich Wichmann, Inke R. König, Andreas Ziegler, Florian Kronenberg, Nilesh J. Samani, Heribert Schunkert, for the Wellcome Trust Case Control Consortium (WTCCC) and the Cardiogenics Consortium

**Affiliations:** 1 Medizinische Klinik II, Universität zu Lübeck, Lübeck, Germany; 2 Institut für Medizinische Biometrie und Statistik, Universität zu Lübeck, Lübeck, Germany; 3 Department of Cardiovascular Sciences, Glenfield Hospital, University of Leicester, Leicester, United Kingdom; 4 Klinik und Poliklinik für Innere Medizin II, Universität Regensburg, Regensburg, Germany; 5 Institut für Klinische Molekularbiologie, Christian-Albrechts Universität, Kiel, Germany; 6 Bundesforschungsanstalt für Ernährung und Lebensmittel, Institut für Physiologie und Biochemie der Ernährung, Kiel, Germany; 7 Klinik für Psychiatrie und Psychotherapie des Kindes- und Jugendalters, Rheinische Kliniken Essen, Universität Duisburg-Essen, Essen, Germany; 8 Vestische Kinder- und Jugendklinik, Universität Witten/Herdecke, Datteln, Germany; 9 Zentrum für Kinderheilkunde der Universität Bonn, Bonn, Germany; 10 Children's Hospital & Regional Medical Centre, University of Washington, Seattle, Washington, United States of America; 11 Klinik für Innere Medizin, Rheinisch-Westfälische Technische Hochschule, Aachen, Germany; 12 C-NET Group, Leeds Institute for Genetics and Therapeutics, Faculty of Medicine and Health, University of Leeds, Leeds, United Kingdom; 13 Institute of Epidemiology, Helmholtz Zentrum München, German Research Center for Environmental Health, Neuherberg, Germany; 14 Institute of Human Genetics, Helmholtz Zentrum München, German Research Center for Environmental Health, Neuherberg, Germany; 15 Department of Medical Genetics, Molecular and Clinical Pharmacology, Medical University Innsbruck, Innsbruck, Austria; 16 Institut für Humangenetik, Technische Universität München, München, Germany; Peninsula Medical School, United Kingdom

## Abstract

**Background:**

Rare mutations of the low-density lipoprotein receptor gene (*LDLR*) cause familial hypercholesterolemia, which increases the risk for coronary artery disease (CAD). Less is known about the implications of common genetic variation in the *LDLR* gene regarding the variability of cholesterol levels and risk of CAD.

**Methods:**

Imputed genotype data at the LDLR locus on 1 644 individuals of a population-based sample were explored for association with LDL-C level. Replication of association with LDL-C level was sought for the most significant single nucleotide polymorphism (SNP) within the *LDLR* gene in three European samples comprising 6 642 adults and 533 children. Association of this SNP with CAD was examined in six case-control studies involving more than 15 000 individuals.

**Findings:**

Each copy of the minor T allele of SNP rs2228671 within *LDLR* (frequency 11%) was related to a decrease of LDL-C levels by 0.19 mmol/L (95% confidence interval (CI) [0.13–0.24] mmol/L, p = 1.5×10^−10^). This association with LDL-C was uniformly found in children, men, and women of all samples studied. In parallel, the T allele of rs2228671 was associated with a significantly lower risk of CAD (Odds Ratio per copy of the T allele: 0.82, 95% CI [0.76–0.89], p = 2.1×10^−7^). Adjustment for LDL-C levels by logistic regression or Mendelian Randomisation models abolished the significant association between rs2228671 with CAD completely, indicating a functional link between the genetic variant at the *LDLR* gene locus, change in LDL-C and risk of CAD.

**Conclusion:**

A common variant at the *LDLR* gene locus affects LDL-C levels and, thereby, the risk for CAD.

## Introduction

A causal link between serum cholesterol levels and coronary artery disease (CAD) risk is supported by multiple epidemiological association studies as well as treatment trials involving lipid-lowering drugs[1]. Less clear are the implications of acquired versus heritable variability of low-density lipoprotein cholesterol (LDL-C) levels as the latter may come into effect already during childhood[2]. Some insights in this respect come from relatively rare mutations either decreasing or increasing LDL-C levels. For example, a variant in the *PCSK9* gene has been associated with a substantial decrease in serum LDL-C levels and almost abolished myocardial infarction (MI) risk[3]. Similarly, rare mutations in the LDL-receptor gene (*LDLR*) are known to cause familial hypercholesterolemia (FH), a condition which is characterized by markedly increased LDL-C levels resulting in premature manifestation of atherosclerotic disease[4]. More recently, several genome wide association studies on lipid traits have identified common SNPs located in the LDLR gene locus showing significant association with LDL-C levels[5], [6], [7]. The study by Willer and coworkers has also presented suggestive evidence for the association of SNP rs6517720 with CAD on one population [6]. This SNP shows high linkage disequilibrium with SNP rs2228671 for which the association with LDL-C and CAD is investigated in detail and across multiple populations in the study presented here.

Inheritance of either relatively low or high LDL-C levels may be interpreted as a Mendelian Randomisation study, which allows to specifically analyse the genetic component in the variability of this risk factor[8], [9]. Particularly, such studies offer the opportunity to test a potentially causal association between risk factor and disease given that interference of behavioural, social, and environmental factors, that otherwise may confound the relationship, is largely excluded by “Randomisation to the genetic variant”. Here, we attempted to identify more frequent genetic variants affecting LDL-C levels and thereby to further study the relation between the inherited variability of serum cholesterol and CAD risk in the European population.

## Methods

### Study design

**Figure 1:**
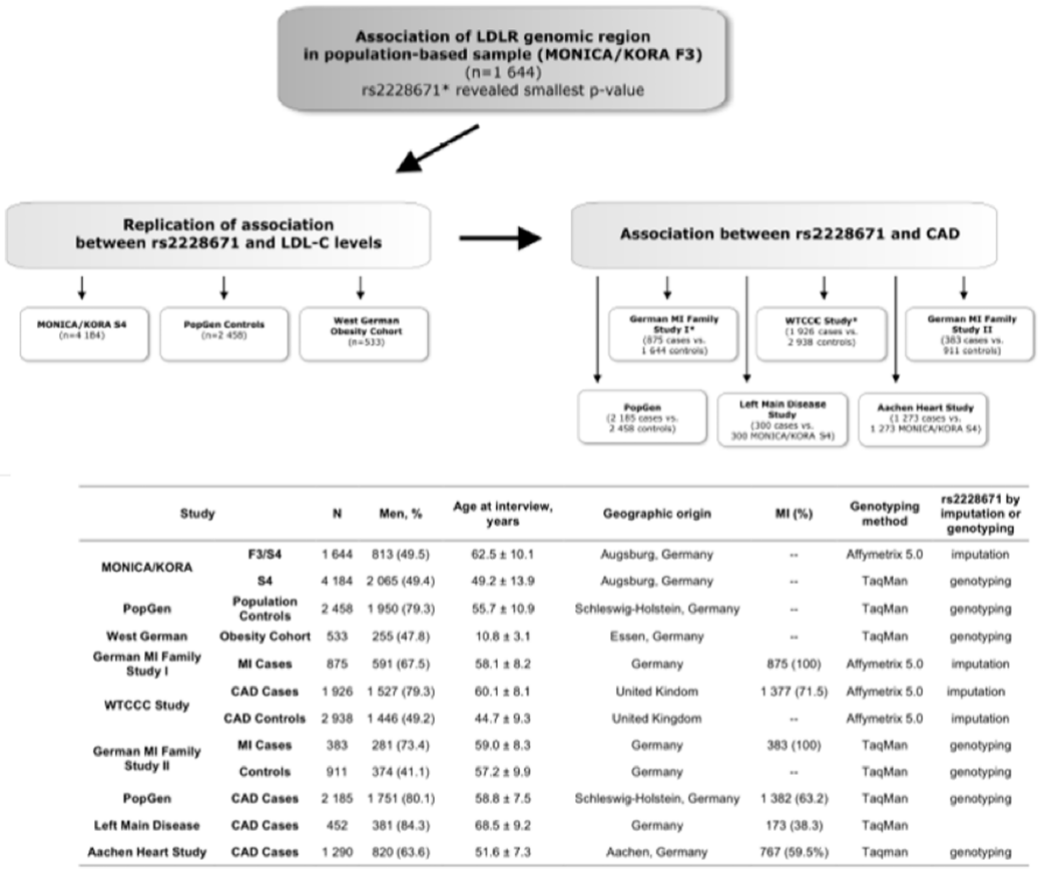
Figure 1a: Strategy for exploring the association of *LDLR* polymorphism rs2228671 with LDL-C levels and CAD across multiple populations. Studies based on imputed genotypes for rs2228671 are marked with a *. Figure 1b: Schematic overview of study populations

Initially, the LDLR genomic region was screened in 1644 population-based subjects of the KORA F3 study for association with LDL levels using genotype data obtained with the GeneChip® Human Mapping 500K Array (Affymetrix, ) enriched by imputed genotypes based on the genome-wide dataset. The single nucleotide polymorphism (SNP) at the *LDLR* gene locus showing the strongest association with LDL-C levels was further investigated. Particularly, association with LDL-C was examined in several population-based studies comprising adults and children, and association with coronary artery disease (CAD) was tested in six case-control studies. Figure 1 a and 1b provide an overview of the experimental strategy and study populations involved. Details on each of the study populations are provided in the supplementary methods (Methods S1 and table S1). Briefly, the MONICA/KORA F3 and S4 studies as well as the PopGen population controls represent individuals from strictly age- and gender-stratified surveys of the German population[10], [11], [12], [13]. A total of 533 overweight but otherwise healthy children and adolescents (mean age 10.8±3.1 years) came from the West German Obesity Cohort of the Bonn/Datteln Childhood Obesity Study Group [14].

To investigate the association of SNP rs2228671 with CAD we performed genotyping of this SNP in four case-control studies (German MI Family Study II[15], PopGen CAD cases[12], Left Main Disease Study[16], Aachen Heart Study[17]) and imputed genotypes for this SNP in two further studies for which genome-wide association data were available (German MI Family Study I, WTCCC CAD study)[18]. Control subjects from the KORA F3 and S4 samples were matched to cases for the German MI Family study 1, Left-main disease and Aachen Heart studies. The matching procedure ensured that a control subject was used only once in any of these three samples. No LDL-values were available for the subjects of the Aachen, WTCCC and Left-main disease studies. All samples have been established in accordance with the principles of the Declaration of Helsinki and were approved by the respective local ethics committees. Laboratory measurements of LDL-C in non-fasting individuals were performed after precipitation of apolipoprotein-B containing lipoproteins and measured with standard enzymatic methods. Gene arrays in the MONICA/KORA F3 survey, the WTCCC CAD study and the German MI Family Study I were performed with the Affymetrix GeneChip® 500K Mapping Array Set as previously described in detail[18]. For the MONICA/KORA S4, PopGen, German MI Family Study II studies and West German Obesity Cohort genotyping for rs2228671 was done using 5′exonuclease activity (Taqman) assay on a HT7900 (Applied Biosystems, Darmstadt, Germany). SNP-assays were ordered from Applied Biosystems as Custom TaqMan® SNP genotyping assay.

### Statistical analysis

At the *LDLR* locus (Chromosome 19p13.2, base pair position 10.900.000 to 11.300.000 according to NCBI Build 36.1), we investigated 33 genotyped and 223 imputed SNPs for association with LDL-C levels. For assessing the effects of variants not represented on the Affymetrix GeneChip® 500K Mapping Array Set, haplotypes were estimated using the CEU sample from the HapMap project (http://www.hapmap.org/)[19] and further variants were imputed using MACH 1.0 (http://www.sph.umich.edu/csg/abecasis/MACH/index.html). Association analysis of LDL-C on SNPs adjusted for age and gender were performed with MACH2QTL. SNPs with a predicted coefficient of determination r^2^<0.3 were excluded from the analyses.

Allele frequencies were analyzed in control populations by a χ^2^-test.

For every study group individually, deviations of genotype distribution from and compatibility with Hardy-Weinberg equilibrium (HWE) were analyzed (details see supplement).

LDL-C was regressed on rs2228671 for each population-based group and subjects of the West German Obesity Cohort separately. A prospectively planned pooled analysis using individual data as proposed by Blettner et al. [20] was performed using all control groups studied by regressing LDL-C on rs2228671 with study as random effect (details see supplement). We additionally stratified the analysis by gender and age groups. Means, standard errors, and p-values are reported.

The power for detecting an association between rs2228671 and CAD was calculated using Quanto (http://hydra.usc.edu/GxE/) assuming a log-additive model α = 0.05 and power = 80% (details see supplement).

Differences in genotype frequencies of rs2228671 were compared between CAD cases and controls for every study using the two-sided asymptotic Cochrane-Armitage trend test. Heterozygous and homozygous odds ratios (OR) for the minor allele and corresponding 95% confidence intervals (CI) were estimated. In the prospectively planned pooled analysis[20] we investigated the additive effect of rs2228671 on CAD unadjusted and adjusted for LDL-C by random effect logistic regression models. The most likely genetic model for rs2228671 on serum LDL-C and for rs2228671 on CAD was investigated using logistic regression models and the Minelli approach[21] (details see supplement)^16^, ^22^ .

There was no adjustment for population stratification (details see supplement).

The association of rs2228671 with serum LDL-C was utilized to investigate the possible causal effect of serum LDL-C on CAD (Mendelian Randomisation). The inheritance of rs2228671 should be subject to the random assortment of parental alleles at the time of gamete formation. If serum LDL-C increases the risk of CAD, then carriage of an allele that exposes individuals to a long-term elevation in serum LDL-C should confer an increased risk of CAD proportional to the difference in serum LDL-C attributable to the allele. This relationship is largely unconfounded and free of reverse causality bias if specific assumptions are met [23], [24], [25], [26], [27] e.g., that rs2228671 is independent of CAD given serum LDL-C. To investigate these relationships between LDL-C, CAD and rs2228671, we fitted a structural equation model using data from cases and controls with known non-use of lipid lowering agents or estimated before-treatment LDL-C levels by correction of measured values for the dosage of a given lipid lowering agent according to a previously published algorithm [28]. In addition, structural equation models including known confounders (age, gender, smoking status, diabetes, and hypertension) were fitted. In case of non-linear variables, the structural equation approach allows a simple interpretation of the regression coefficients when compared with an instrumental variable approach [5]. Estimation was done using MPlus 5, which allows specification of categorical variables, and sensitivity analyses were performed with SEPATH using correlation coefficients estimated by use of the polychor package within R. For this analysis, adjustments for population stratification in the present studies are not required because only German Caucasian subjects were included in this analysis, and population heterogeneity is negligible in the German population as previously demonstrated [Samani et al. 2007; Steffens et al. 2006].

## Results

**Figure 2:**
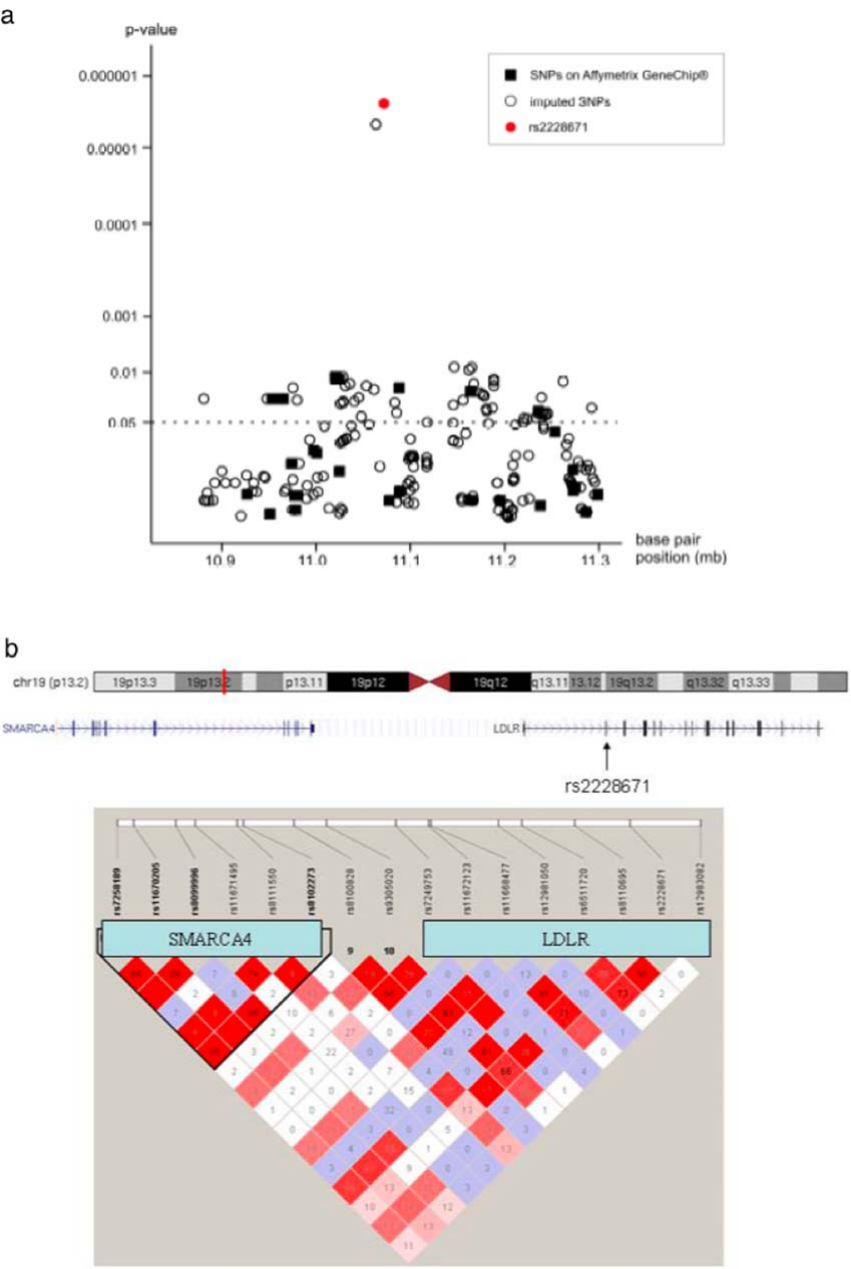
Figure 2a: Association of the chromosomal locus 19p13.2 with LDL-C levels in population-based sample. Presented are p-values from linear regression of LDL-C on SNPs adjusted for age and gender. p-values of the SNPs genotyped on the Affymetrix GeneChip® 500K Mapping Array Set are displayed as closed squares, p-values predicted by HapMap-based imputation are displayed as blank circles. SNP rs2228671 is highlighted as a red circle. Figure 2b: Linkage disequilibrium structure of the genomic region surrounding SNP rs2228671. Displayed are the HapMap data (r^2^;[19]) of all SNPs surrounding rs2228671 (11030947–11079189) and the genes within this region.

The genomic region of the *LDLR* located at 19p13.2 was screened for association with LDL-C levels using data from a gene array study performed in the MONICA/KORA F3 survey. Nine of 33 SNPs representing the region on the Affymetrix GeneChip® 500K Mapping Array Set (black squares in Fig. 2a) showed association with LDL-C levels with a p-value <0.05 (see table S2). A higher density of SNPs mapping to the *LDLR* genomic region resulted from imputation of further SNPs, which predicted genotypes for 223 additional SNPs located at 19p13.2. Of these, 185 passed quality analyses (blank circles in Fig. 2a; see Methods section), and 50 were associated with LDL-C levels with a p-value <0.05. Two of these SNPs showed markedly higher levels of significance for association with LDL-C than the surrounding SNPs: rs6511720 (predicted p-value = 4.6×10^−6^) and rs2228671 (predicted p-value = 2.4×10^−6^). The SNP with the strongest effect (0.36 mmol/L mean difference per one T allele, 95% CI [0.21,0.52] mmol/L; rs2228671; red circle in Fig. 2a) was chosen for replication in further populations.

**Figure 3:**
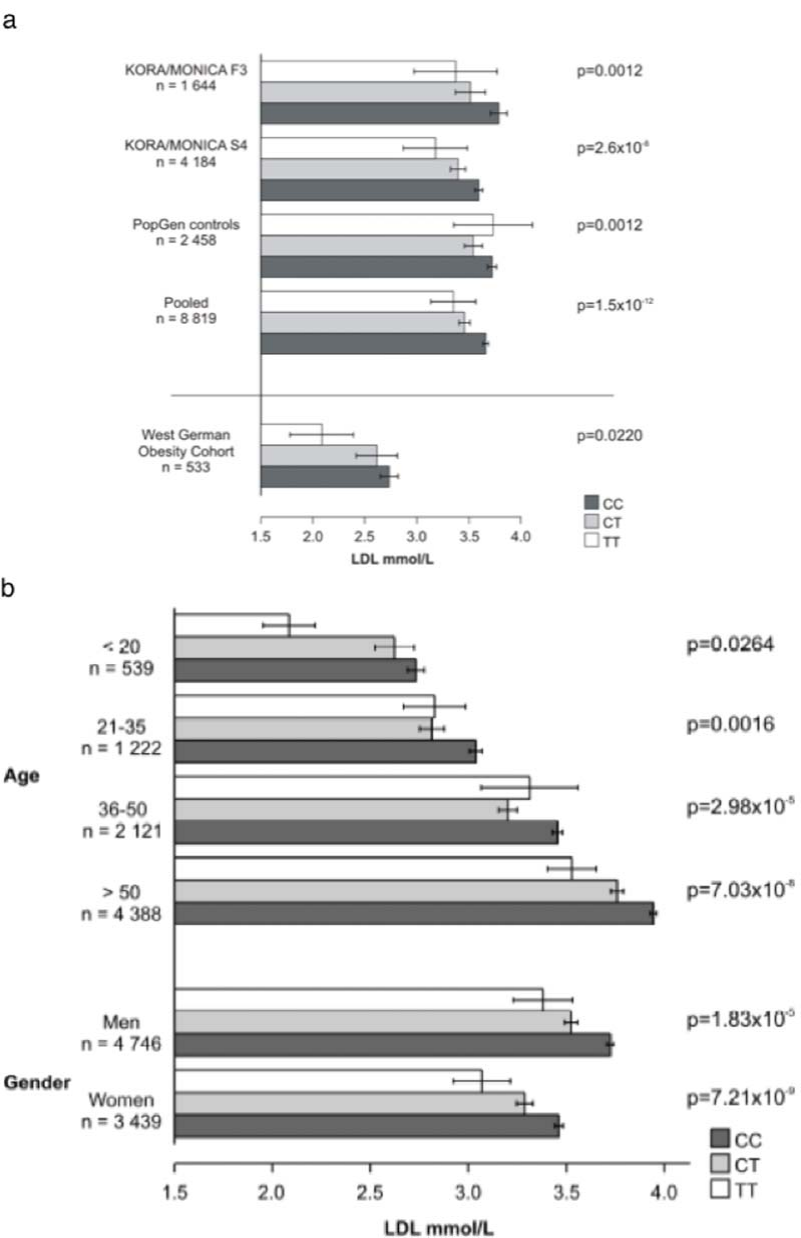
Figure 3a: Association of SNP rs2228671 with LDL-C levels across populations. Displayed are means of LDL-C and standard error of means stratified by rs2228671 genotype. Unadjusted levels of significance are displayed for each individual study. Effects for the pooled study are from a random effect linear regression model. Figure 3b: Pooled analysis of the association of rs2228671 with LDL-C across populations stratified by age groups and gender. Displayed are means of LDL-C and standard error of means stratified by rs2228671 genotype, separately for age groups (<20, 21–35, 36–50, >50), men and women.

In order to verify the predicted association of the imputation experiment we genotyped SNP rs2228671 using the 5′-exonuclease method in 1 644 subjects of the MONICA/KORA F3 study demonstrating a slightly higher (3.6%) minor allele frequency than in imputed genotypes. However, the relation between rs2228671 and LDL-C remained consistent for the genotyped SNP (Fig. 3a). We then confirmed this association in the larger MONICA/KORA S4 study comprising 4 184 individuals with a mean difference per T allele of 0.20 mmol/L (95% CI [0.13–0.28] mmol/L; p = 2.6×10^−8^) and the population-based PopGen study comprising 2 458 individuals with a mean difference per T allele of 0.14 mmol/L (95% CI [0.06–0.23] mmol/L; p = 0.0012; Fig. 3a).

In order to test if the association of rs2228671 with LDL-C was already detectable before adulthood we performed additional genotyping in children and adolescents referred for treatment of obesity. In this sample we found a mean difference per T allele of 0.20 mmol/L (95% CI [0.03–0.37] mmol/L; p = 0.0220; Fig. 3a).

The genetic effect of rs2228671 on LDL-C is most likely additive (see Methods S1). This implies that individuals carrying a copy of the T allele have a lifelong exposure to lower LDL-C levels than homozygous C allele carriers with a reduction of 0.19 mmol/L per T allele (95% CI [0.13–0.24] mmol/L; p = 1.5×10^−12^; Fig. 3a). Furthermore, the genetic variant represented by SNP rs2228671 affected LDL-C levels to a similar extent in men and women and across different age groups (Fig. 3b).

**Figure 4:**
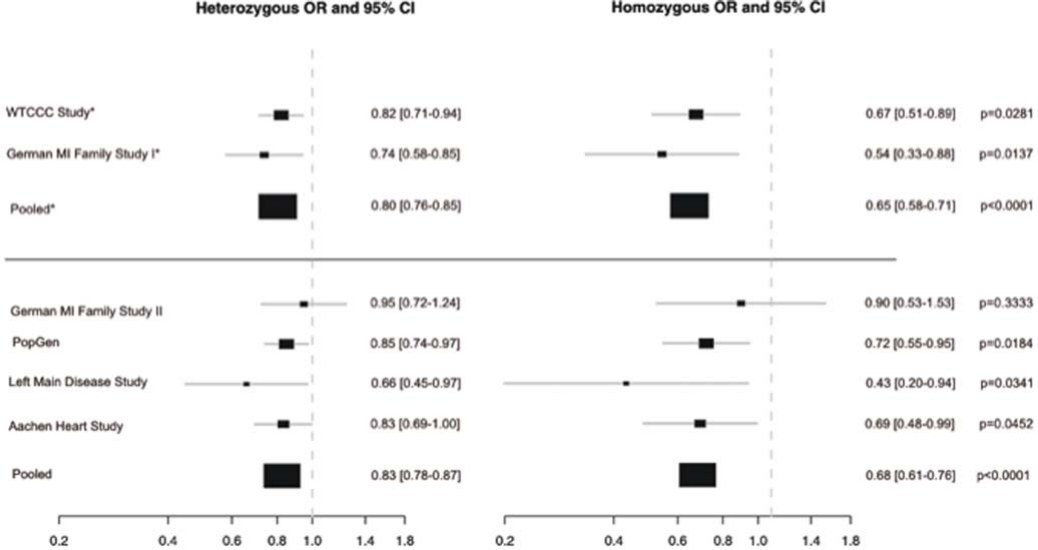
Association of rs2228671 with risk of CAD in six case-control studies. Displayed are heterozygous and homozygous ORs for CAD and 95% CI per study from two-sided asymptotic Cochrane-Armitage trend test. Pooled ORs, 95% CIs and p-values are from random effect logistic regression models. The number of individuals in each study is represented by size of the closed square. SNP rs2228671 was imputed from the Affymetrix GeneChip® 500K Mapping Array Set in the WTCCC and German MI Family Study I and genotyped directly in the remaining studies.

Since carriers of the minor allele of SNP rs2228671 have a lifetime of lower LDL-C levels we hypothesized that this translates into a lower CAD risk. To explore this hypothesis, we performed imputation of SNPs located at the *LDLR* gene locus in two genome-wide association studies of the German MI Family I and WTCCC CAD studies (Fig. 1). In both studies we observed a significant association of the imputed T allele of rs2228671 with CAD risk resulting, expectedly, in decreased ORs for disease manifestation (Fig. 4). Additionally, the association of rs2228671 with CAD was explored by genotyping in four case-control studies for CAD comprising a total number of 4 310 cases and 8 086 controls. Across all six studies, we found an association with CAD with an OR per copy of the T allele of 0.82 (95 % CI [0.76–0.89], p = 2.1×10^−7^, Fig. 4, for genotype distribution and p-values of individual studies see Table S3).

According to the PROCAM-Algorithm[29] a reduction in LDL-C of 0.38 mmol/L, as found in TT versus CC homozygous individuals in the population-based studies translates into a 21% reduction in the risk of cardiovascular events over a 10 year period. In fact, reduction in risk of CAD by 23% was observed for homozygosity of the minor allele of rs2228671 in the combined analysis of all individuals.

**Figure 5:**
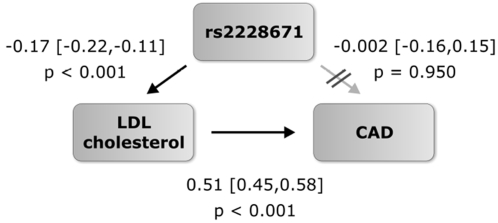
Results of Mendelian Randomisation study. Exploration of the causal relationship between LDL-C associated with rs2228671 and CAD (Mendelian Randomisation). In the structural equation model, carriage of the T allele at rs2228671 leads to lower LDL-C levels, and higher LDL-C levels lead to an increased risk of CAD. Given this, there is no additional direct path from rs2228671 to CAD risk, indicating that the functional pathway between the genetic variant at the *LDLR* gene locus and risk of CAD is through changes in LDL-C.

In order to further examine whether a lower risk of CAD, as observed in carriers of the T allele, is causally linked to the genetically determined lower LDL-C associated with this common SNP in the *LDLR* gene, we performed a Mendelian Randomisation study. For this analysis we focused on individuals on whom native LDL-C levels, i.e., levels not a ffected by or corrected for lipid lowering medication, were available or could be estimated (1 324 cases and 6 255 controls). The structural equation model revealed a highly significant association between the genotype and LDL-C as well as between LDL-C and CAD risk, but the association between rs2228671 and the risk of CAD was abolished (OR 0.99, 95 % CI [0.85–1.16], p = 0.95, Fig. 5). Including known confounders, which were found to be independent of rs2228671, did not change the results (see Fig. S1). Further, these findings were confirmed by regression analysis adjusting the CAD risk related to rs2228671 for LDL-C (see Table S3). Thus, the protective effect on CAD risk observed for the T allele is likely to be causally linked to the reduction in LDL-C associated with this particular variant in the *LDLR* gene (Fig. 5).

## Discussion

We identified a common variant in the *LDLR* gene that is related to lower LDL-C levels and a lower risk of CAD and MI. In the PROCAM and Framingham risk scores, a decrease of LDL-C by 0.38 mmol/L, as found in homozygote carriers of the T allele, matches a 21–23% decrease of MI risk[29], [30]. We observed an effect of such magnitude in six consecutive samples with CAD cases and healthy controls.

We replicated the association of rs2228671 with LDL-C levels in individuals across a wide age spectrum including children. Interestingly, the magnitude of difference in LDL-C levels was stable in all subgroups studied suggesting a stepwise decrease of lifetime exposure to lower cholesterol levels for each copy of the T allele. In parallel, we observed a stepwise decrease in CAD risk. Together with the data from the Mendelian Randomisation, the findings plausibly suggest a functional link between the decrease in cholesterol and the decrease in CAD risk related to the genetic variant or a closely linked variant.

From a clinical point of view the variability in LDL-C modulated by rs2228671 genotypes might be considered rather small in comparison to the profound LDL-C lowering achieved in recent therapeutic trials (examples: CARE[31]: Δ1.1 mmol/L, Heart Protection Study[32]: Δ1.0 mmol/L,TNT[33]: Δ1.9 mmol/L )[34]. Yet the risk reduction for CAD events reached almost the same magnitude as observed by statin treatment in these trials[1]. Thus, it seems conceivable that a lifelong reduction of LDL-C by 0.38 mmol/L in homozygotes for the T rather than the C allele or in individuals followed long-term in epidemiological cohorts is comparable to the effects of 3–5 years of statin treatment, which by itself results in much larger LDL-C lowering. In this respect it may be of inerest that the variability of LDL-C levels carries a high heritability such that the effects observed in the population may indeed reflect to a large extent the effects of underlying genetic factors[35]. Previous studies using the Mendelian Randomization approach have observed an even greater change in CAD risk through lifelong genetic lowering of cholesterol than predicted by epidemiological scores [2], [3]. However, the sample size of the rare variants was small such that the conclusions of these previous studies on PCSK9 are in line with our study with the risk reduction observed for the protective allele being similar to the risk reduction predicted by epidemiological scores.

Several genes are known to affect LDL-C and MI risk including those coding for *APOB* [36], [37], *LDLR* [38], and *PCSK9* [3]. However, until recently only variants with fairly low allele frequencies ranging between 0.001 to 0.08% in the *APOB* gene [36], 0.01 to 0.02% in the *LDLR* gene [39], and 2 to 3 % in the PCSK9 gene had been identified [3]. With the availability of GWAs based DNA-Arrays, a number of more frequent variants affecting LDL-levels emerged [5], [6], [7], [35], [40]. Indeed, association of rs2228671 with LDL-C can be found in the comprehensive data of a GWA meta-analysis of three British populations comprising 4337 individuals[5]. Furthermore, the SNP has previously been implicated with hyperlipidemia in interaction models and haplotype analyses[41], [42]. However, a direct association of this SNP with LDL-C levels and CAD risk has not been demonstrated so far. While it was unclear whether the T allele of the *LDLR* polymorphism rs2228671 also affects coronary artery disease risk the relatively high prevalence of the minor allele (frequency 11%) made it plausible that the contribution of rs2228671 to the variability of LDL-C serum levels also translates to a modification in CAD risk at the population level.

In parallel to the present work, Kathiresan and coworkers studied two polymorphisms from the LDLR locus (rs1529729 and rs688) in a CAD prediction score that was based on genotypes that had previously displayed association with lipid levels. Indeed, this score allowed to predict cardiovascular events in a prospective cohort [35]. However, both SNPs from the LDLR included in this score are not in significant linkage disequilibrium with rs2228671 such that the present findings are likely to further extend the implications of common genetic variation for prediction of LDL variability and CAD risk.

Strengths of the present study include the utilisation of several population-based samples and several cohort studies displaying consistently significant effects across multiple sub-groups as well as broad replication in multiple case-control studies. Moreover, evidence for a causal link between modulation of LDL-C levels and CAD risk is strongly supported by the Mendelian Randomisation approach. Nevertheless, limitations of the present work need consideration including the fact that we cannot mechanistically explain the decrease in LDL-C associated with the T-allele. SNP rs228671 is located within the first exon of *LDLR* in the third position of a triplet coding for amino acid 27, a cysteine (Fig. 2b). However, allelic variation in this SNP does not result in an amino acid change. No further polymorphisms in *LDLR* are known to cause amino acid changes or previously associated with LDL-C levels or CAD were found to be in linkage disequilibrium with rs2228671 or rs6511720[6], [7], suggesting that hitherto unknown mechanisms are responsible for this association. Both, gain of function or increased expression of the *LDLR* are possible to affect LDL-C serum levels. Remotely, there is even a chance that another gene in linkage disequilibrium with the variant accounts for the observed effects.

Whatever the underlying molecular mechanism is, from a genetic point of view this study on multiple populations offers robust data that the rs2228671 T-allele goes along with lower LDL-C levels and, secondary to this effect, a lower risk of CAD.

## Supporting Information

Methods S1 (0.13 MB DOC)

Table S1 (0.07 MB DOC)

Table S2 (0.09 MB DOC)

Table S3 (0.05 MB DOC)

Table S4 (0.04 MB DOC)

Figure S1 (0.58 MB DOC)

## References

[1] Grundy SM, Cleeman JI, Merz CN, Brewer HB, Clark LT (2004). Implications of recent clinical trials for the National Cholesterol Education Program Adult Treatment Panel III guidelines.. Circulation.

[2] Brown MS, Goldstein JL (2006). Biomedicine. Lowering LDL–not only how low, but how long?. Science.

[3] Cohen JC, Boerwinkle E, Mosley TH, Hobbs HH (2006). Sequence variations in PCSK9, low LDL, and protection against coronary heart disease.. N Engl J Med.

[4] Brown MS, Goldstein JL (1974). Expression of the familial hypercholesterolemia gene in heterozygotes: mechanism for a dominant disorder in man.. Science.

[5] Sandhu M, Waterworth DM, Debenham SL, Wheeler W, Papadakis K (2008). LDL-cholesterol concentrations: a genome-wide association study.. Lancet.

[6] Willer CJ, Sanna S, Jackson AU, Scuteri A, Bonnycastle LL (2008). Newly identified loci that influence lipid concentrations and risk of coronary artery disease.. Nat Genet.

[7] Kathiresan S, Melander O, Guiducci C, Surti A, Burtt NP (2008). Six new loci associated with blood low-density lipoprotein cholesterol, high-density lipoprotein cholesterol or triglycerides in humans.. Nat Genet.

[8] Lawlor DA, Harbord RM, Sterne JA, Timpson N, Davey Smith G (2008). Mendelian randomization: Using genes as instruments for making causal inferences in epidemiology.. Stat Med.

[9] Ziegler A, Pahlke F, Konig IR (2008). Comments on ‘Mendelian randomization: using genes as instruments for making causal inferences in epidemiology’ by Debbie A. Lawlor, R. M. Harbord, J. A. Sterne, N. Timpson and G. Davey Smith, Statistics in Medicine, DOI: 10.1002/sim.3034.. Stat Med.

[10] Wichmann HE, Gieger C, Illig T (2005). KORA-gen–resource for population genetics, controls and a broad spectrum of disease phenotypes.. Gesundheitswesen.

[11] Meisinger C, Heier M, Doering A, Thorand B, Loewel H (2004). Prevalence of known diabetes and antidiabetic therapy between 1984/1985 and 1999/2001 in southern Germany.. Diabetes Care.

[12] Krawczak M, Nikolaus S, von Eberstein H, Croucher PJ, El Mokhtari NE (2006). PopGen: population-based recruitment of patients and controls for the analysis of complex genotype-phenotype relationships.. Community Genet.

[13] Rubin D, Helwig U, Pfeuffer M, Schreiber S, Boeing H (2006). A common functional exon polymorphism in the microsomal triglyceride transfer protein gene is associated with type 2 diabetes, impaired glucose metabolism and insulin levels.. J Hum Genet.

[14] Reinehr T, Hinney A, de Sousa G, Austrup F, J H (2007). Definable somatic disorders in overweight children and adolescents.. Journal of Pediatrics.

[15] Lieb W, Mayer B, Konig IR, Borwitzky I, Gotz A (2008). Lack of association between the MEF2A gene and myocardial infarction.. Circulation.

[16] Schunkert H, Gotz A, Braund P (2008). Repeated Replication and Meta Analysis of the Association Between Chromosome 9p21.3 and Coronary Artery Disease.. Circulation in the press.

[17] Ortlepp JR, von Korff A, Hanrath P, Zerres K, Hoffmann R (2003). Vitamin D receptor gene polymorphism BsmI is not associated with the prevalence and severity of CAD in a large-scale angiographic cohort of 3441 patients.. Eur J Clin Invest.

[18] Samani NJ, Erdmann J, Hall AS, Hengstenberg C, Mangino M (2007). Genomewide association analysis of coronary artery disease.. N Engl J Med.

[19] Frazer KA, Ballinger DG, Cox DR, Hinds DA, Stuve LL (2007). A second generation human haplotype map of over 3.1 million SNPs.. Nature.

[20] Blettner M, Sauerbrei W, Schlehofer B, Scheuchenpflug T, Friedenreich C (1999). Traditional Reviews, Meta-Analyses and Pooled Analyses in Epidemiology.. Int J Epidemiol.

[21] Minelli C, Thompson JR, Abrams KR, Thakkinstian A, Attia J (2005). The choice of a genetic model in the meta-analysis of molecular association studies.. Int J Epidemiol.

[22] Bagos PG, Nikolopoulos GK (2007). A method for meta-analysis of case-control genetic association studies using logistic regression.. Stat Appl Genet Mol Biol.

[23] Davey Smith G, Ebrahim S (2003). ‘Mendelian randomisation’: can genetic epidemiology contribute to understanding environmental determinants of disease?. International Journal of Epidemiology.

[24] Thomas DC, Conti DV (2004). Commentary: the concept of ‘Mendelian Randomization’.. International Journal of Epidemiology.

[25] Bautista LE, Smeeth L, Hingorani AD, Casas JP (2006). Estimation of bias in nongenetic observational studies using ‘Mendelian triangulation’.. Annals of Epidemiology.

[26] Thomas DC, Lawlor DA, Thompson JR (2007). Re: estimation of bias in nongenetic observational studies using ‘Mendelian triangulation’ by Bautista et al.. Annals of Epidemiology.

[27] Davey Smith G, Ebrahim S (2004). Mendelian randomization: prospects, potentials, and limitations.. International Journal of Epidemiology.

[28] Baessler A, Fischer M, Huf V, Mell S, Hengstenberg C (2005). Failure to achieve recommended LDL cholesterol levels by suboptimal statin therapy relates to elevated cardiac event rates.. Int J Cardiol.

[29] Assmann G, Cullen P, Schulte H (2002). Simple scoring scheme for calculating the risk of acute coronary events based on the 10-year follow-up of the prospective cardiovascular Munster (PROCAM) study.. Circulation.

[30] Wilson PW, D'Agostino RB, Levy D, Belanger AM, Silbershatz H (1998). Prediction of coronary heart disease using risk factor categories.. Circulation.

[31] Lewis SJ, Sacks FM, Mitchell JS, East C, Glasser S (1998). Effect of pravastatin on cardiovascular events in women after myocardial infarction: the cholesterol and recurrent events (CARE) trial.. J Am Coll Cardiol.

[32] (2002). MRC/BHF Heart Protection Study of cholesterol lowering with simvastatin in 20,536 high-risk individuals: a randomised placebo-controlled trial.. Lancet.

[33] LaRosa JC, Grundy SM, Waters DD, Shear C, Barter P (2005). Intensive lipid lowering with atorvastatin in patients with stable coronary disease.. N Engl J Med.

[34] Lewington S, Whitlock G, Clarke R, Sherliker P, Emberson J (2007). Blood cholesterol and vascular mortality by age, sex, and blood pressure: a meta-analysis of individual data from 61 prospective studies with 55,000 vascular deaths.. Lancet.

[35] Kathiresan S, Melander O, Anevski D, Guiducci C, Burtt NP (2008). Polymorphisms associated with cholesterol and risk of cardiovascular events.. N Engl J Med.

[36] Tybjaerg-Hansen A, Steffensen R, Meinertz H, Schnohr P, Nordestgaard BG (1998). Association of mutations in the apolipoprotein B gene with hypercholesterolemia and the risk of ischemic heart disease.. N Engl J Med.

[37] Benn M, Stene MC, Nordestgaard BG, Jensen GB, Steffensen R (2007). Common and Rare Alleles in Apolipoprotein B Contribute to Plasma Levels of LDL Cholesterol in the General Population.. J Clin Endocrinol Metab.

[38] Zhu H, Tucker HM, Grear KE, Simpson JF, Manning AK (2007). A common polymorphism decreases low-density lipoprotein receptor exon 12 splicing efficiency and associates with increased cholesterol.. Hum Mol Genet.

[39] (2001). The Metabolic and Molecular Bases of Inherited Disease. 4th edition..

[40] Wallace C, Newhouse JC, Braund P, Zhang F, Tobin MD (2008). Genome-wide Association Study Identifies Genes for Biomarkers of Cardiovascular Disease: Serum Urate and Dyslipidemia.. Am J Hum Genet.

[41] Costanza MC, Cayanis E, Ross BM, Flaherty MS, Alvin GB (2005). Relative contributions of genes, environment, and interactions to blood lipid concentrations in a general adult population.. Am J Epidemiol.

[42] Bauerfeind A, Knoblauch H, Costanza MC, Luganskaja T, Toliat MR (2006). Concordant association of lipid gene variation with a combined HDL/LDL-cholesterol phenotype in two European populations.. Hum Hered.

