# Correction: Lifelong Reduction of LDL-Cholesterol Related to a Common Variant in the LDL-Receptor Gene Decreases the Risk of Coronary Artery Disease—A Mendelian Randomisation Study

**DOI:** 10.1371/annotation/9f64c41a-8cf6-40f2-8988-0d48b04dd8cb

**Published:** 2008-09-03

**Authors:** Patrick Linsel-Nitschke, Anika Götz, Jeanette Erdmann, Ingrid Braenne, Peter Braund, Christian Hengstenberg, Klaus Stark, Marcus Fischer, Stefan Schreiber, Nour Eddine El Mokhtari, Arne Schaefer, Jürgen Schrezenmeir, Diana Rubin, Anke Hinney, Thomas Reinehr, Christian Roth, Jan Ortlepp, Peter Hanrath, Alistair S. Hall, Massimo Mangino, Wolfgang Lieb, Claudia Lamina, Iris M. Heid, Angela Doering, Christian Gieger, Annette Peters, Thomas Meitinger, H.-Erich Wichmann, Inke R. König, Andreas Ziegler, Florian Kronenberg, Nilesh J. Samani, Heribert Schunkert, for the Wellcome Trust Case Control Consortium (WTCCC) and the Cardiogenics Consortium

The twelfth author's name was spelled incorrectly. It should read: Jürgen Schrezenmeir.

