# Supplementary material for: Lifelong Reduction of LDL-Cholesterol Related to a Common Variant in the LDL-Receptor Gene Decreases the Risk of Coronary Artery Disease—A Mendelian Randomisation Study
Source: PLoS One. 2008 Aug 20;3(8):e2986. doi: 10.1371/journal.pone.0002986 (PMC2500189; doi:10.1371/journal.pone.0002986)
Supplement: Methods S1 — (0.13 MB DOC) [file pone.0002986.s001.doc]

# Supporting information on Methods

**Details of study populations:**

**KORA S3, F3, S4**

The third **(S3)** and fourth survey **(S4)** and the Follow-up study **(F3)** of the population-based (Monitoring of Trends and Determinations of Cardiovascular Disease) / KORA (Cooperative Research in the Region of Augsburg) studies were conducted between 1994 - 1995 and 1999 – 2001 and 2004-2005 respectively. The **KORA** project was carried out as a component of the international collaborative WHO project[1]. It investigated the cardiovascular risk factor profile of randomly selected subjects of the Augsburg population in cross-sectional surveys. The study design, sampling frame, and data collection have been described in detail elsewhere[2,3]. A sub-sample of KORA F3 including 1644 subjects (KORA F3 500K project) was genotyped with the GeneChip® Human Mapping 500K Array (Affymetrix).

# PopGen Population Controls

Healthy controls for this sample were identified through official population registries of the state of Schleswig-Holstein, the northernmost region in Germany. Participants were first contacted by telephone and then provided with a standardized questionnaire. Briefly, a total of 7 200 individuals with 2 400 people in each of three age groups (18-30, 30-50, 50-80 years) were recruited for the study. Of these, a random sample of 2 458 individuals was genotyped. Details of the recruitment process have been described elsewhere [4,5].

# West German Obesity Cohort

This cohort comprises 533 German overweight and obese but otherwise healthy children and adolescents, age spectrum 1 to 18 years, mean age 10.8 ± 3.1 years (mean BMI 28.9 ± 5.4 kg/m^2^). The children were either recruited at the Department of Paediatrics, University of Bonn, Germany (n = 194) or at the `Vestische Kinderklinik Datteln´, University of Witten/Herdecke, Germany (n = 339). The ascertainment strategy was previously described in detail[6].

# German MI Family Study I and II

The German Myocardial Infarction (MI) Family Study case samples I (n = 875) and II (n = 383) consisted of individuals with premature MI (before age of 66 years) from families with at least two first-degree relatives affected by premature MI and/or premature severe CAD defined by percutaneous coronary intervention or coronary bypass grafting[7]. The majority of families were identified in cardiac rehabilitation clinics in the vicinity of Augsburg. All events were validated through inspection of hospital charts. Only one subject from each family was selected for genotyping, either the youngest male MI patient, or preferentially, an affected female MI patient. Control subjects (n = 1 644) for German MI I were Caucasians of German origin who participated in the population based /KORA Augsburg S3/F3 survey. Healthy married in spouses (n = 911) served as control subjects for the German MI Family II study.

# WTCCC CAD Study

Cases (n = 1 926) had a validated history of either MI or coronary revascularisation (coronary artery bypass surgery or percutaneous coronary angioplasty) before their 66^th^ birthday as well as a strong familial basis of coronary artery disease (CAD). All were of Caucasian descent and were recruited from throughout the United Kingdom (UK)[8]. 1 500 controls (UK Blood Services (UKBS)) were selected from a sample of blood donors recruited as part of the WTCCC project. A second sample of 1 500 controls derived from the 1958-Birth Cohort Study. The control subjects were about equally divided into males and females. Like the case collection, individuals of the control cohort were geographically widely distributed across the UK. The UKBS controls had a wide age range with the majority of subjects between 40 - 59 years[9].

**PopGen CAD cases**

This study included 2 185 unrelated German CAD patients who were recruited in Schleswig-Holstein, the northernmost region in Germany, through the population-based PopGen biobank. Coronary angiograms of any of the five cardiac catheterization laboratories in this geographical area were screened. Study subjects were required to have coronary catheterization demonstrating significant CAD (at least a 70% stenosis in one major epicardial coronary vessel) before the age of 55 years. All patients were between 34 and 64 years of age at the time of recruitment and younger than 55 years at the time of disease onset. PopGen controls were matched by ancestry (all Germans from Schleswig-Holstein), age, and sex and are described above.

## Left Main Disease Study

For this study, out of approximately 10 000 patients undergoing coronary angiography at the University of Lübeck hospital between the years 2001 until 2005 we have retrospectively identified 300 unrelated patients with significant left-main disease, a coronary phenotype with high heritability[10]. Left main disease was defined as luminal narrowing of the left main stem larger than 50%. Age- (± 5 years) and sex-matched healthy individuals from KORA/ S4 served as control subjects.

**Aachen Heart Study**

This study included 1 290 unrelated subjects with significant (> 50%) narrowing of a major epicardial vessel documented on coronary angiography in males below 55 or females below 65 years of age. Additionally, all cases had a documented history of ischemia-related symptoms and/or positive stress testing, subjects with non-ischemic cardiomyopathy or valvular heart disease were excluded from the study. Details of the recruiment process have been described elsewhere[11]. Age- (± 3 years) and sex-matched healthy individuals from KORA/ S4 served as control subjects.

**Details of Lipid measurements:**

Laboratory measurements of LDL-C were performed in non-fasting individuals and measured with standard enzymatic methods: (CHOD-PAP) after precipitation with dextrane sulfate in the supernatant, except for KORA F3 in which LDL cholesterol was measured with direct methods (AHDL Flex and ALDL Flex, Dade Behring).

**Details of statistical methods:**

**Quality control:**

Stringent quality controls were used on all levels. Imputation SNPs with a predicted r < 0.3 were excluded from the analyses. In total, 38 SNPs had to be excluded because of r < 0.3 from the analyses.

For the genotyped data, for every study group individually, deviations of genotype distribution from and compatibility with Hardy-Weinberg equilibrium (HWE) were analyzed using a Monte Carlo χ^2^ goodness-of-fit test and the exact uniformly most powerful equivalence test with δ_2_ = 2 and δ_1_ = 2/3[12,13].

# Beside the West German Obesity Cohort, rs2228671 conformed with HWE in all control and case study groups. The number of heterozygotes in the West German Obesity Cohort is 69, but should be according to the equivalence test between 75 and 79. The deviation from HWE in this study population at this SNP is potentially caused by the ascertainment strategy of selecting for obese children and adolescents.

# Homogeneity of genotype frequencies between the control groups of the different studies was checked with the asymptotic χ^2^ test of independence. Allele frequencies in the genotyped control groups were homogeneous (p = 0.1311).

**Prospectively planned pooled analysis on LDL:**

A prospectively planned pooled analysis using individual data as proposed by Blettner et al.[14] was performed using all control groups studied by regressing LDL on rs2228671. We investigated the additive effect of rs2228671 on CAD by random effect (RE) linear regression model with adjustment for study. In the RE approach, inferences are to be generalized to a population in which the studies are permitted to have different effects and different characteristics. This is plausible in this investigation, because the different studies vary in their inclusion and exclusion criteria. Specifically, we fitted a RE linear regression model with RE for study and fixed effect (FE) for genotype. These analyses were conducted using SAS (version 9.1.3, SP 4, SAS Institute, Inc.; proc mixed). A standard linear regression model with FEs for both study and genotype was used as sensitivity analysis.

**Prospectively planned pooled analysis on CAD:**

This study was also planned as a prospective pooled analysis according to Blettner et al.[14]. Separately for genotyped and imputed data, we performed RE logistic regression models on CAD with an additive FE of rs2228671 and a RE of study.

In addition, the logistic regression framework was also used to identify the underlying genetic model. Specifically, we used an ordinal coding with two dummy variables and tested the hypotheses of a) a recessive, b) a dominant and c) an additive mode of inheritance using FE for the genotype and a RE for the study. The goodness of fit in this approach was determined using the Akaike information criterion. The logistic regression framework to identify the underlying genetic model rejected an autosomal-dominant (p < 0.0001) as well as an autosomal-recessive mode of inheritance (each p = 0.0030). The most likely model is an additive genetic model because it is the only genetic model not rejected at 5% test level (p = 0.4934). Analyses were performed using SAS (version 9.1.3, SP 4, SAS Institute, Inc.; proc gee). A standard logistic regression model with FEs for both study and genotype was used as sensitivity analysis.

To identify the genetic model, we second followed Minelli et al.[15] and estimated the ratio of the log odds ratio for the heterozygous individuals compared to homozygous individuals, i.e. λ equals 0, 1/2, 1, and >1 if the genetic model is recessive, additive, dominant, and positive heterosis, respectively. To obtain 95% confidence intervals for λ, we used a nonparametric bootstrap with 10 000 replicates. Although the 95% confidence interval [0.15,1.40] in our study is rather wide, the point estimator $\hat{\lambda}$ = 0.49 strongly supports an additive genetic model.

Finally, we used the aggregated estimators of every study to pool all imputed and genotyped studies using the DerSimonian and Laird approach[16].

**Population stratification:**

In a prior study we extensively investigated population stratification in the entire German population[17]. The genetic differentiation is low between North and South Germany (Fst = 1.7x10^-4^) and between East and South Germany (Fst = 5.4x10^-4^). Such minor degree of population substructure cannot be detected from methods without using prior information of subpopulation membership.

With respect to the present study, both cases and controls were ascertained within small catchments areas. Furthermore, an inflation factor of l=0.998 on the outcome variable LDL-C was calculated using the GWA results of imputed genotypes (~2.3 million SNPs) in the KORA F3 sample. We thus consider that population sub-structure is negligible in our studies and unlikely to explain the associations observed. We nevertheless adjusted for the recruitment centre, because differences in inclusion and exclusion criteria are most likely more relevant than population stratification.

The inflation factor estimated in the previously published genome-wide association study[8] was 1.02 in the German population, and the 95% confidence interval clearly covered 1.00.

**Power analysis:**

1. Details on the power analysis are provided in table S4. The power for detecting an association between rs2228671 and CAD was calculated as suggested by Gauderman[18] which is implemented in Quanto (hydra.usc.edu/GxE/).
2. We assumed a log-additive model and α=0.05. Given the unknown true underlying effect, we varied odds ratios from 0.5 to 0.9. Allele frequencies in the case-control studies were assumed based on the allele frequencies from the LDL-C replication studies (KORA/ F3: 10.6%, KORA/ S4: 11.7%, PopGen Controls: 11.2%, West German Obesity Cohort: 9.6%) and therefore were varied between 8% and 12%. Given is the number of case-control pairs required for power of 80% assuming the different odds ratios and allele frequencies.
3. We finally evaluated a total of 4 310 cases and 8 086 controls. Thus we had at least 80% power to detect an odds ratio of 0.9 at a SNP with an allele frequency of 8%.

**References for supplementary material:**

1. (1988) The World Health Organization MONICA Project (monitoring trends and determinants in cardiovascular disease): a major international collaboration. WHO MONICA Project Principal Investigators. J Clin Epidemiol 41: 105-114.

2. Meisinger C, Heier M, Doering A, Thorand B, Loewel H (2004) Prevalence of known diabetes and antidiabetic therapy between 1984/1985 and 1999/2001 in southern Germany. Diabetes Care 27: 2985-2987.

3. Wichmann HE, Gieger C, Illig T (2005) KORA-gen--resource for population genetics, controls and a broad spectrum of disease phenotypes. Gesundheitswesen 67: S26-S30.

4. Krawczak M, Nikolaus S, von Eberstein H, Croucher PJ, El Mokhtari NE, et al. (2006) PopGen: population-based recruitment of patients and controls for the analysis of complex genotype-phenotype relationships. Community Genet 9: 55-61.

5. Rubin D, Helwig U, Pfeuffer M, Schreiber S, Boeing H, et al. (2006) A common

functional exon polymorphism in the microsomal triglyceride transfer

protein gene is associated with type 2 diabetes, impaired glucose

metabolism and insulin levels. J Hum Genet 51: 567-574.

6. Reinehr T, Hinney A, de Sousa G, Austrup F, J H, et al. (2007) Definable somatic disorders in overweight children and adolescents. Journal of Pediatrics 150: 618-622.

7. Broeckel U, Hengstenberg C, Mayer B, Holmer S, Martin LJ, et al. (2002) A comprehensive linkage analysis for myocardial infarction and its related risk factors. Nat Genet 30: 210-214.

8. Samani NJ, Erdmann J, Hall AS, Hengstenberg C, Mangino M, et al. (2007) Genomewide association analysis of coronary artery disease. N Engl J Med 357: 443-453.

9. (2007) Genome-wide association study of 14,000 cases of seven common diseases and 3,000 shared controls. Nature 447: 661-678.

10. Fischer M, Broeckel U, Holmer S, Baessler A, Hengstenberg C, et al. (2005) Distinct heritable patterns of angiographic coronary artery disease in families with myocardial infarction. Circulation 111: 855-862.

11. Ortlepp JR, von Korff A, Hanrath P, Zerres K, Hoffmann R (2003) Vitamin D receptor gene polymorphism BsmI is not associated with the prevalence and severity of CAD in a large-scale angiographic cohort of 3441 patients. Eur J Clin Invest 33: 106-109.

12. Ziegler A KI (2006) A Statistical Approach to Genetic Epidemiology. Concepts and Applications.

13. Wellek S (2004) Tests for establishing compatibility of an observed genotype distribution with Hardy-Weinberg equilibrium in the case of a biallelic locus. Biometrics 60: 694-703.

14. Blettner M, Sauerbrei W, Schlehofer B, Scheuchenpflug T, Friedenreich C (1999) Traditional Reviews, Meta-Analyses and Pooled Analyses in Epidemiology. Int J Epidemiol 28: 1-9.

15. Minelli C, Thompson JR, Abrams KR, Thakkinstian A, Attia J (2005) The choice of a genetic model in the meta-analysis of molecular association studies. Int J Epidemiol 34: 1319-1328.

16. DerSimonian R, Laird N (1986) Meta-analysis in clinical trials. Control Clin Trials 7: 177-188.

17. Steffens M, Lamina C, Illig T (2006) SNP-based analysis of genetic substructure in the German population. Human Heredity 62: 20-29.

18. Gaudermann W (2002) Sample size requirements for matched case-control studies of gene-environment interaction. Stat Med 21: 35-50.

**List of members of the WTCCC and Cardiogenics consortia:**

### WTCCC Consortium

Management Committee: Paul R Burton^1^, David G Clayton^2^, Lon R Cardon^3^, Nick Craddock^4^, Panos Deloukas^5^, Audrey Duncanson^6^, Dominic P Kwiatkowski^3,5^, Mark I McCarthy^3,7^, Willem H Ouwehand^8,9^, Nilesh J Samani^10^, John A Todd^2^, Peter Donnelly (Chair)^11^

Analysis Committee: Jeffrey C Barrett^3^, Paul R Burton^1^, Dan Davison^11^, Peter Donnelly^11^, Doug Easton^12^, David Evans^3^, Hin-Tak Leung^2^, Jonathan L Marchini^11^, Andrew P Morris^3^, Chris CA Spencer^11^, Martin D Tobin^1^, Lon R Cardon (Co-chair)^3^, David G Clayton (Co-chair)^2^

UK Blood Services & University of Cambridge Controls: Antony P Attwood^5,8^, James P Boorman^8,9^, Barbara Cant^8^, Ursula Everson^13^, Judith M Hussey^14^, Jennifer D Jolley^8^, Alexandra S Knight^8^, Kerstin Koch^8^, Elizabeth Meech^15^, Sarah Nutland^2^, Christopher V Prowse^16^, Helen E Stevens^2^, Niall C Taylor^8^, Graham R Walters^17^, Neil M Walker^2^, Nicholas A Watkins^8,9^, Thilo Winzer^8^, John A Todd^2^, Willem H Ouwehand^8,9^

1958 Birth Cohort Controls: Richard W Jones^18^, Wendy L McArdle^18^, Susan M Ring^18^, David P Strachan^19^, Marcus Pembrey^18,20^

Coronary Artery Disease (Leeds): Stephen G Ball^21^, Anthony J Balmforth^21^, Jennifer H Barrett^21^, D Timothy Bishop^21^, Mark M Iles^21^, Azhar Maqbool^21^, Nadira Yuldasheva^21^, Alistair S Hall^21^; (Leicester): Peter S Braund^10^, Paul R Burton^1^, Richard J Dixon^10^, Massimo Mangino^10^, Suzanne Stevens^10^, Martin D Tobin^1^, John R Thompson^1^, Nilesh J Samani^10^

DNA, Genotyping, Data QC and Informatics (Wellcome Trust Sanger Institute, Hinxton): Suzannah J Bumpstead^5^, Amy Chaney^5^, Kate Downes^2,5^, Mohammed JR Ghori^5^, Rhian Gwilliam^5^, Sarah E Hunt^5^, Michael Inouye^5^, Andrew Keniry^5^, Emma King^5^, Ralph McGinnis^5^, Simon Potter^5^, Rathi Ravindrarajah^5^, Pamela Whittaker^5^, Claire Widden^5^, David Withers^5^, Panos Deloukas^5^; (Cambridge): Hin-Tak Leung^2^, Sarah Nutland^2^, Helen E Stevens^2^, Neil M Walker^2^, John A Todd^2^

Statistics (Cambridge): Doug Easton^12^, David G Clayton^2^; (Leicester): Paul R Burton^1^, Martin D Tobin^1^; (Oxford): Jeffrey C Barrett^3^, David Evans^3^, Andrew P Morris^3^, Lon R Cardon^3^; (Oxford): Niall J Cardin^11^, Dan Davison^11^, Teresa Ferreira^11^, Joanne Pereira-Gale^11^, Ingeleif B Hallgrimsdóttir^11^, Bryan N Howie^11^, Jonathan L Marchini^11^, Chris CA Spencer^11^, Zhan Su^11^, Yik Ying Teo^3,11^, Damjan Vukcevic^11^, Peter Donnelly^11^

^1^ Genetic Epidemiology Group, Department of Health Sciences, University of Leicester, Adrian Building, University Road, Leicester, LE1 7RH, UK; ^2^ Juvenile Diabetes Research Foundation/Wellcome Trust Diabetes and Inflammation Laboratory, Department of Medical Genetics, Cambridge Institute for Medical Research, University of Cambridge, Wellcome Trust/MRC Building, Cambridge, CB2 0XY, UK; ^3^ Wellcome Trust Centre for Human Genetics, University of Oxford, Roosevelt Drive, Oxford OX3 7BN, UK; ^4^ Department of Psychological Medicine, Henry Wellcome Building, School of Medicine, Cardiff University, Heath Park, Cardiff CF14 4XN, UK; ^5^ The Wellcome Trust Sanger Institute, Wellcome Trust Genome Campus, Hinxton, Cambridge CB10 1SA, UK; ^6^ The Wellcome Trust, Gibbs Building, 215 Euston Road, London NW1 2BE, UK; ^7^ Oxford Centre for Diabetes, Endocrinology and Medicine, University of Oxford, Churchill Hospital, Oxford, OX3 7LJ, UK; ^8^ Department of Haematology, University of Cambridge, Long Road, Cambridge, CB2 2PT, UK; ^9^ National Health Service Blood and Transplant, Cambridge Centre, Long Road, Cambridge, CB2 2PT, UK; ^10^ Department of Cardiovascular Sciences, University of Leicester, Glenfield Hospital, Groby Road, Leicester, LE3 9QP, UK; ^11^ Department of Statistics, University of Oxford, 1 South Parks Road, Oxford OX1 3TG, UK; ^12^ Cancer Research UK Genetic Epidemiology Unit, Strangeways Research Laboratory, Worts Causeway, Cambridge CB1 8RN, UK^; 13^ National Health Service Blood and Transplant, Sheffield Centre, Longley Lane, Sheffield S5 7JN, UK; ^14^ National Health Service Blood and Transplant, Brentwood Centre, Crescent Drive, Brentwood, CM15 8DP, UK; ^15^ The Welsh Blood Service, Ely Valley Road, Talbot Green, Pontyclun, CF72 9WB, UK; ^16^ The Scottish National Blood Transfusion Service, Ellen’s Glen Road, Edinburgh, EH17 7QT, UK; ^17^ National Health Service Blood and Transplant, Southampton Centre, Coxford Road, Southampton, SO16 5AF, UK; ^18^ Avon Longitudinal Study of Parents and Children, University of Bristol, 24 Tyndall Avenue, Bristol, BS8 1TQ, UK; ^19^ Division of Community Health Services, St George’s University of London, Cranmer Terrace, London SW17 0RE, UK; ^20^ Institute of Child Health, University College London, 30 Guilford St, London WC1N 1EH, UK; ^21^ LIGHT and LIMM Research Institutes, Faculty of Medicine and Health, University of Leeds, Leeds, LS1 3EX, UK.

Cardiogenics Consortium

Associazione per lo studio dell Trombosi in Cardiologia, 27100 Pavia, Italy: Diego Ardissino

EUROIMMUN AG, 23560 Lübeck, Germany: Christian Krüger, Lars Komorowski, Christian Probst, Ulf Steller

GSF GmbH, 85764 Neuherberg, Germany: Thomas Meitinger, Erich Wichmann, Peter Lichtner

Institut National de la Santé et de la Recherche Médicale (INSERM), 75654 Paris, France: Francois Cambien, Laurence Tiret, Florent Soubrier

Johannes Gutenberg-University of Mainz, 55099 Mainz, Germany: Stefan Blankenberg, Karl Lackner, Thomas Münzel

Queen’s University of Belfast, Belfast BT71NN, UK: Alun Evans, Frank Kee

Sanquin Blood Supply Foundation, 1066 CX Amsterdam, The Netherlands: Ellen van der Schoot, Jaap Jan Zwaginga

Technische Universität München, 80333 München, Germany: Dietlind Zohlnhoefer

TRIUM Analysis Online GmbH, 81677 München, Germany: Martin Daumer, Michael Scholz

The Chancellor, Master and Scholar of the University of Cambridge, Cambridge CB2 1TS, UK: Willem Ouwehand, Richard Farndale, Nicholas Watkins

University of Leeds, Leeds LS2 9JT Leeds, UK: Alistair Hall, Stephen Ball, Tim Bishop

University of Leicester, Leicester LE1 7RH, UK: Nilesh Samani, Alison Goodall, Paul Burton

University of Lübeck, 23538 Lübeck, Germany: Heribert Schunkert, Jeanette Erdmann, Andreas Ziegler

University of Regensburg, 93053 Regensburg, Germany: Christian Hengstenberg, Gerd Schmitz

University of Uppsala, 75105 Uppsala, Sweden: Ann-Christin Syvänen, Tomas Axelsson, Ulrika Liljedahl

Wellcome Trust Sanger Institute, Hinxton Cambridge CB10 1SA, UK: Panos Deloukas, Jane Rogers, Kate Rice
