# Supplementary material for: Lifelong Reduction of LDL-Cholesterol Related to a Common Variant in the LDL-Receptor Gene Decreases the Risk of Coronary Artery Disease—A Mendelian Randomisation Study
Source: PLoS One. 2008 Aug 20;3(8):e2986. doi: 10.1371/journal.pone.0002986 (PMC2500189; doi:10.1371/journal.pone.0002986)
Supplement: Table S1 — (0.07 MB DOC) [file pone.0002986.s002.doc]

**Table S1a:** Baseline characteristics of the study populations.

| **Study** | | **N** | **Men, %** | **Age at interview,  years** | **Age of onset,  years** | **MI (%)** | **SBP, mm HG** | **DBP, mm HG** |
| --- | --- | --- | --- | --- | --- | --- | --- | --- |
| **/KORA** | **F3/S4** | 1 644 | 813 (49.5) | 62.5 ± 10.1 | -- | -- | -- | -- |
|  | **S4** | 4 184 | 2 065 (49.4) | 49.2 ± 13.9 | -- | -- | -- | -- |
| **PopGen** | **Population Controls** | 2 458 | 1 950 (79.3) | 55.7 ± 10.9 | -- | -- | 133.5 ± 17.8 | 82.7 ± 10.2 |
| **West German** | **Obesity Cohort** | 533 | 255 (47.8) | 10.8 ± 3.1 | -- | -- | -- | -- |
| **German MI Family Study I** | **MI Cases** | 875 | 591 (67.5) | 58.1 ± 8.2 | 50.2 ± 7.9 | 875 (100) | 138.8 ± 20.3 | 82.8 ± 10.3 |
| **WTCCC Study** | **CAD Cases** | 1 926 | 1 527 (79.3) | 60.1 ± 8.1 | 49.8 ± 7.7 | 1 377 (71.5) | 143.0 ± 23.7 | 84.1 ± 12.9 |
|  | **CAD Controls** | 2 938 | 1 446 (49.2) | 44.7 ± 9.3 | -- | -- | -- | -- |
| **German MI Family Study II** | **MI Cases** | 383 | 281 (73.4) | 59.0 ± 8.3 | 50.4 ± 7.8 | 383 (100) | 136.3 ± 20.4 | 81.7 ± 10.3 |
|  | **Controls** | 911 | 374 (41.1) | 57.2 ± 9.9 | -- | -- | 134.3 ± 17.5 | 82.2 ± 9.9 |
| **PopGen** | **CAD Cases** | 2 185 | 1 751 (80.1) | 58.8 ± 7.5 | -- | 1 382 (63.2) | -- | -- |
| **Left Main Disease** | **CAD Cases** | 452 | 381 (84.3) | 68.5 ± 9.2 | 60.5 ± 11.6 | 173 (38.3) | 135.1 ± 18.6 | 79.2 ± 11.0 |
| **Aachen Heart Study** | **CAD Cases** | 1 290 | 820 (63.6) | 51.6 ± 7.3 | -- | 767 (59.5%) | -- | -- |

| **Study** | | **cholesterol,  mmol/L** | **HDL cholesterol,  mmol/L** | **LDL cholesterol,  mmol/L** | **Smoking (%)** | **Diabetes (%)** | **Statins (%)** |
| --- | --- | --- | --- | --- | --- | --- | --- |
| **/KORA** | **F3/S4** | 5.7 ± 1.0 | 1.5 ± 0.4 | 3.4 ± 0.8 | -- | -- | 243 (14.8) |
|  | **S4** | 5.9 ± 1.1 | 1.5 ± 0.4 | 3.6 ± 1.1 | 2 379 (56.9) | 164 (3.9) | 247 (5.9) |
| **PopGen** | **Population Controls** | 5.7 ± 1.1 | 1.6 ± 0.5 | 3.7 ± 1.0 | 1 726 (70.2) | 84 (3.4) | 4 (0.2) |
| **West German** | **Obesity Cohort** | -- | 1.3 ± 0.3 | 2.7 ± 0.8 | -- | -- | -- |
| **German MI Family Study I** | **MI Cases** | 5.9 ± 1.2 | 1.3 ± 0.3 | 4.0 ± 1.1 | 615 (70.3) | 108 (12.3) | 575 (65.7) |
| **WTCCC Study** | **CAD Cases** | 5.3 ± 1.0 | 1.2 ± 0.4 | 3.1 ± 0.9 | 1 460 (75.8) | 211 (11.0) | 1 369 (71.1) |
|  | **CAD Controls** | -- | -- | -- | -- | -- | -- |
| **German MI Family Study II** | **MI Cases** | 5.8 ± 1.2 | 1.3 ± 0.4 | 3.8 ± 1.1 | 268 (70.0) | 60 (15.7) | 236 (61.6) |
|  | **Controls** | 6.2 ± 1.1 | 1.6 ± 0.4 | 3.8 ± 0.9 | 463 (50.8) | 53 (5.8) | 79 (8.7) |
| **PopGen** | **CAD Cases** | 5.5 ± 1.4 | 1.2 ± 0.5 | 3.6 ± 1.2 | 1 654 (75.7) | 426 (19.5) | 1 778 (81.4) |
| **Left Main Disease** | **CAD Cases** | 5.9 ± 1.9 | 1.3 ± 0.4 | 3.9 ± 1.1 | 123 (27.2) | 104 (23.0) | -- |
| **Aachen Heart Study** | **CAD Cases** | -- | -- | -- | 915 (70.9) | 241 (18.7) | -- |

Table S1a: Baseline characteristics of the study populations.

MI indicates myocardial infarction; SBP indicates systolic blood pressare; DBP indicates diastolic blood pressure; BMI indicates body mass index;

diabetes was defined by fasting glucose above 125mg/dl or treatment with insulin or oral antidiabetics or anamnestic; receiving lipid-lowering drugs means receiving statins or fibrates (in WTCCC only receiving statins).

**Table S1b: LDL-C concentrations in control populations stratified by genotype**

| Study | CC | | | CT | | | TT | | |
| --- | --- | --- | --- | --- | --- | --- | --- | --- | --- |
|  | mean | ± | stdev | mean | ± | stdev | mean | ± | stdev |
| KORA F3 | 3.79 | ± | 1.15 | 3.52 | ± | 1.02 | 3.37 | ± | 0.66 |
| KORA S4 | 3.60 | ± | 1.07 | 3.40 | ± | 1.06 | 3.18 | ± | 1.16 |
| PopGen controls | 3.73 | ± | 0.93 | 3.55 | ± | 0.95 | 3.74 | ± | 0.92 |
| West German Obesity Cohort | 2.74 | ± | 0.80 | 2.62 | ± | 0.79 | 2.09 | ± | 0.40 |

Displayed are means and standard deviations (stdev) of LDL-C concentrations (mmol/L) stratified by genotype.
