# Supplementary material for: Lifelong Reduction of LDL-Cholesterol Related to a Common Variant in the LDL-Receptor Gene Decreases the Risk of Coronary Artery Disease—A Mendelian Randomisation Study
Source: PLoS One. 2008 Aug 20;3(8):e2986. doi: 10.1371/journal.pone.0002986 (PMC2500189; doi:10.1371/journal.pone.0002986)
Supplement: Table S2 — (0.09 MB DOC) [file pone.0002986.s003.doc]

**Table S2:** Imputed associations with LDL levels in KORA/ F3 study

| **rs ID** | **position** | **500K** | **Minor Allele** | **MAF** | **r** | **p-value** | **Effect [95% CI]** |
| --- | --- | --- | --- | --- | --- | --- | --- |
| rs11667234 | 10880581 |  | A | 3.7 | 0.77 | 0.024 | 0.19 [0.02,0.35] |
| rs17001070 | 10947746 |  | C | 3.5 | 0.92 | 0.024 | 0.19 [0.02,0.35] |
| rs11669133 | 10953139 | yes | A | 3.5 | 0.93 | 0.024 | 0.18 [0.02,0.35] |
| rs17001072 | 10960775 | yes | G | 3.5 | 0.93 | 0.024 | 0.18 [0.02,0.35] |
| rs17001074 | 10965121 | yes | T | 3.5 | 0.93 | 0.024 | 0.18 [0.02,0.34] |
| rs12983316 | 10975352 |  | G | 17.1 | 0.44 | 0.017 | 0.14 [0.02,0.25] |
| rs17001090 | 10979711 |  | T | 3.5 | 0.97 | 0.025 | 0.18 [0.02,0.34] |
| rs12052201 | 11020096 | yes | T | 26.0 | 1.02 | 0.012 | -0.08 [-0.15,-0.02] |
| rs12052058 | 11020525 | yes | T | 25.9 | 1.01 | 0.013 | -0.08 [-0.15,-0.02] |
| rs3786722 | 11022537 |  | A | 26.0 | 1.01 | 0.012 | -0.08 [-0.15,-0.02] |
| rs1122608 | 11024601 | yes | T | 25.9 | 1.01 | 0.013 | -0.08 [-0.15,-0.02] |
| rs6511719 | 11025790 |  | G | 3.2 | 0.58 | 0.028 | 0.20 [0.02,0.38] |
| rs12609863 | 11028219 |  | T | 25.9 | 0.93 | 0.012 | -0.09 [-0.16,-0.02] |
| rs7245660 | 11028495 |  | G | 3.2 | 0.54 | 0.027 | 0.21 [0.02,0.39] |
| rs17001114 | 11028581 |  | A | 3.2 | 0.54 | 0.026 | 0.21 [0.02,0.39] |
| rs7258189 | 11030947 |  | C | 32.8 | 0.68 | 0.016 | -0.09 [-0.16,-0.02] |
| rs8099996 | 11035625 |  | G | 32.8 | 0.66 | 0.015 | -0.09 [-0.17,-0.02] |
| rs8111550 | 11040571 |  | T | 2.9 | 0.50 | 0.026 | 0.21 [0.03,0.40] |
| rs8100828 | 11045104 |  | T | 3.7 | 0.50 | 0.023 | 0.20 [0.03,0.37] |
| rs9305020 | 11047711 |  | C | 14.8 | 0.40 | 0.042 | -0.12 [-0.24,0.00] |
| rs7249753 | 11053226 |  | C | 5.3 | 0.47 | 0.016 | 0.19 [0.04,0.34] |
| rs12981050 | 11061412 |  | T | 5.8 | 0.48 | 0.018 | 0.18 [0.03,0.33] |
| rs6511720 | 11063306 |  | T | 8.0 | 0.44 | 4.60E-06 | -0.34 [-0.48,-0.19] |
| rs2228671 | 11071912 |  | T | 7.0 | 0.39 | 2.40E-06 | -0.36 [-0.52,-0.21] |
| rs11669576 | 11083300 |  | A | 7.5 | 0.61 | 0.027 | 0.15 [0.02,0.29] |
| rs5930 | 11085265 |  | A | 41.2 | 0.88 | 0.037 | -0.07 [-0.13,0.00] |
| rs4508523 | 11087944 | yes | T | 14.0 | 0.88 | 0.017 | 0.11 [0.02,0.20] |
| rs10402592 | 11117887 |  | T | 12.4 | 0.33 | 0.05 | 0.14 [0.00,0.27] |
| rs370229 | 11145483 |  | A | 38.1 | 0.63 | 0.029 | 0.08 [0.01,0.16] |
| rs440677 | 11146390 |  | G | 33.5 | 0.53 | 0.0089 | 0.11 [0.03,0.19] |
| rs7253916 | 11155747 |  | A | 38.2 | 0.75 | 0.025 | 0.08 [0.01,0.15] |
| rs8111456 | 11162147 |  | G | 33.1 | 0.85 | 0.0097 | 0.09 [0.02,0.15] |
| rs7249565 | 11163807 | yes | A | 38.3 | 0.96 | 0.019 | 0.07 [0.01,0.13] |
| rs3745681 | 11164943 |  | G | 37.2 | 0.92 | 0.009 | 0.08 [0.02,0.15] |
| rs7250778 | 11167265 |  | A | 37.6 | 0.84 | 0.015 | 0.08 [0.02,0.15] |
| rs3745682 | 11174256 |  | A | 37.4 | 0.78 | 0.02 | 0.08 [0.01,0.15] |
| rs6511727 | 11176817 |  | T | 37.4 | 0.77 | 0.021 | 0.08 [0.01,0.15] |
| rs4804574 | 11178482 |  | G | 37.4 | 0.76 | 0.022 | 0.08 [0.01,0.15] |
| rs8409 | 11180491 |  | G | 37.1 | 0.72 | 0.033 | 0.08 [0.01,0.15] |
| rs11878417 | 11180978 |  | G | 37.1 | 0.72 | 0.035 | 0.08 [0.01,0.15] |
| rs2304155 | 11187119 |  | A | 37.1 | 0.66 | 0.032 | 0.08 [0.01,0.15] |
| rs4804150 | 11188571 |  | C | 48.9 | 0.65 | 0.016 | 0.09 [0.02,0.16] |
| rs4804151 | 11188608 |  | T | 31.2 | 0.55 | 0.013 | 0.11 [0.02,0.19] |
| rs4804152 | 11188626 |  | A | 31.2 | 0.54 | 0.014 | 0.11 [0.02,0.19] |
| rs4804579 | 11219700 |  | C | 17.6 | 0.74 | 0.05 | -0.09 [-0.18,0.00] |
| rs2116876 | 11220484 |  | A | 25.8 | 0.66 | 0.044 | -0.08 [-0.16,0.00] |
| rs8113582 | 11224146 |  | A | 25.9 | 0.67 | 0.045 | -0.08 [-0.16,0.00] |
| rs7248924 | 11233077 |  | C | 17.2 | 0.96 | 0.043 | -0.08 [-0.16,0.00] |
| rs4804582 | 11235555 |  | G | 17.2 | 0.98 | 0.038 | -0.08 [-0.16,0.00] |
| rs416231 | 11235675 | yes | T | 17.2 | 1.00 | 0.035 | -0.08 [-0.16,-0.01] |
| rs322130 | 11238570 |  | C | 17.2 | 0.99 | 0.036 | -0.08 [-0.16,-0.01] |
| rs322131 | 11238588 |  | A | 17.2 | 0.99 | 0.038 | -0.08 [-0.16,0.00] |
| rs322133 | 11238975 |  | A | 13.0 | 0.78 | 0.023 | -0.12 [-0.21,-0.02] |
| rs322135 | 11240717 |  | G | 17.0 | 0.93 | 0.037 | -0.08 [-0.16,0.00] |
| rs638137 | 11242975 |  | C | 16.9 | 0.98 | 0.04 | -0.08 [-0.16,0.00] |
| rs1869772 | 11245207 |  | G | 17.0 | 0.98 | 0.037 | -0.08 [-0.16,-0.01] |
| rs1654403 | 11245407 |  | A | 17.0 | 0.98 | 0.039 | -0.08 [-0.16,0.00] |
| rs322129 | 11262105 |  | T | 22.0 | 0.87 | 0.014 | -0.09 [-0.17,-0.02] |
| rs396115 | 11292566 |  | G | 7.5 | 0.31 | 0.032 | 0.16 [0.01,0.31] |

Presented are results of association analysis of the KORA/ F3 study. Only SNPs with a predicted r > 0.3 were considered for analysis. Displayed are effects (mean difference per minor allele) and corresponding 95% confidence intervals (CI) of SNPs with a p-value < 0.05 in a regression of LDL-C on imputed and genotyped SNPs adjusted for age and gender. Imputation based on MACH 1.0 and analysis were performed with MACH2QTL (see methods). MAF indicates minor allele frequency.
