# Supplementary material for: Lifelong Reduction of LDL-Cholesterol Related to a Common Variant in the LDL-Receptor Gene Decreases the Risk of Coronary Artery Disease—A Mendelian Randomisation Study
Source: PLoS One. 2008 Aug 20;3(8):e2986. doi: 10.1371/journal.pone.0002986 (PMC2500189; doi:10.1371/journal.pone.0002986)
Supplement: Table S3 — (0.05 MB DOC) [file pone.0002986.s004.doc]

**Table S3:** Genotypes and p-values for association with CAD from six case-control studies.

|  |  | **CC** | **CT** | **TT** | **MAF** | **p unadj.** | **OR unadj. [95%CI]** | **p adj.** | **OR adj. [95%CI]** |
| --- | --- | --- | --- | --- | --- | --- | --- | --- | --- |
| German MI  Family Study I* | MI Cases | 781 | 93 | 1 | 5.4 | 0.0281 | 0.75 | n.a. | n.a. |
|  | F3 Controls | 1417 | 224 | 3 | 7.0 |  | [0.59,0.97] |  |  |
| WTCCC* | CAD Cases | 1578 | 322 | 13 | 9.1 | 0.0051 | 0.82 | n.a. | n.a. |
|  | WTCCC Controls | 2332 | 569 | 34 | 10.9 |  | [0.71,0.94] |  |  |
| **Pooled imputed** | **CAD Cases** | **2359** | **415** | **14** | **7.9** | **<0.0001** | **0.80** | n.a. | n.a. |
|  | **Controls** | **3749** | **793** | **37** | **9.5** |  | **[0.77,0.84]** |  |  |
| German MI  Family Study II | MI Cases | 282 | 64 | 3 | 10.0 | 0.3333 | 0.87 | 0.4962 | 0.9 |
|  | Spouses | 671 | 164 | 15 | 11.4 |  | [0.65,1.16] |  | [0.67,1.21] |
| PopGen | CAD Cases | 1755 | 379 | 19 | 9.7 | 0.0184 | 0.85 | 0.6922 | 0.96 |
|  | Controls | 1840 | 474 | 25 | 11.2 |  | [0.74,0.97] |  | [0.80,1.16] |
| Left Main Disease Study | CAD Cases | 236 | 43 | 2 | 8.4 | 0.0343 | 0.66 | 0.9675 | 1.01 |
|  | Matched S4 Controls | 224 | 61 | 5 | 12.2 |  | [0.45,0.97] |  | [0.60,1.70] |
| Aachen Heart  Study | CAD Cases | 937 | 216 | 10 | 10.1 | 0.0453 | 0.83 | n.a. | n.a. |
|  | Matched S4 Controls | 972 | 255 | 22 | 12.0 |  | [0.69,0.99] |  |  |
| **Pooled genotyped** | **CAD Cases** | **3210** | **702** | **34** | **9.8** | **<0.0001** | **0.83** | **0.5003** | **0.95** |
|  | **Controls** | **3707** | **954** | **67** | **11.5** |  | **[0.80,0.86]** |  | **[0.82,1.10]** |
| **Pooled total** | **CAD Cases** | **5569** | **1117** | **48** | **9.0** | 2.1x10^-7^ | **0.82** | n.a. | n.a. |
|  | **Controls** | **7456** | **1747** | **104** | **10.5** |  | **[0.76,0.89]** |  |  |

Displayed are genotype frequencies, minor allele frequencies (MAF) for the two imputed and the four genotyped studies on rs2228671.

Further there are unadjusted p-values and Odds Ratios (ORs) per copy of one T allele from two-sided asymptotic Cochrane-Armitage trend test and LDL-C adjusted p-values and ORs from logistic regression models.

Pooled imputed and genotyped p-values and ORs are from random effect logistic regression model. Pooled total is estimated from the aggregated data of the six single studies (details on pooling see supplementary methods).

n.a. indicates not available.
