# Supplementary material for: Lifelong Reduction of LDL-Cholesterol Related to a Common Variant in the LDL-Receptor Gene Decreases the Risk of Coronary Artery Disease—A Mendelian Randomisation Study
Source: PLoS One. 2008 Aug 20;3(8):e2986. doi: 10.1371/journal.pone.0002986 (PMC2500189; doi:10.1371/journal.pone.0002986)
Supplement: Table S4 — (0.04 MB DOC) [file pone.0002986.s005.doc]

**Table S4:** Power analysis for rs2228671 on CAD.

| **Odds Ratio** | **Allele frequency** | | | | |
| --- | --- | --- | --- | --- | --- |
|  | **0.08** | **0.09** | **0.10** | **0.11** | **0.12** |
| **0.9** | 9 879 | 8 873 | 8 070 | 7 414 | 6 870 |
| **0.8** | 2 280 | 2 046 | 1 860 | 1 708 | 1 582 |
| **0.7** | 931 | 835 | 759 | 697 | 645 |
| **0.6** | 479 | 429 | 390 | 358 | 331 |
| **0.5** | 278 | 250 | 227 | 208 | 192 |
