# Supplementary material for: Lifelong Reduction of LDL-Cholesterol Related to a Common Variant in the LDL-Receptor Gene Decreases the Risk of Coronary Artery Disease—A Mendelian Randomisation Study
Source: PLoS One. 2008 Aug 20;3(8):e2986. doi: 10.1371/journal.pone.0002986 (PMC2500189; doi:10.1371/journal.pone.0002986)
Supplement: Figure S1 — (0.58 MB DOC) [file pone.0002986.s006.doc]

**Figure S1a**


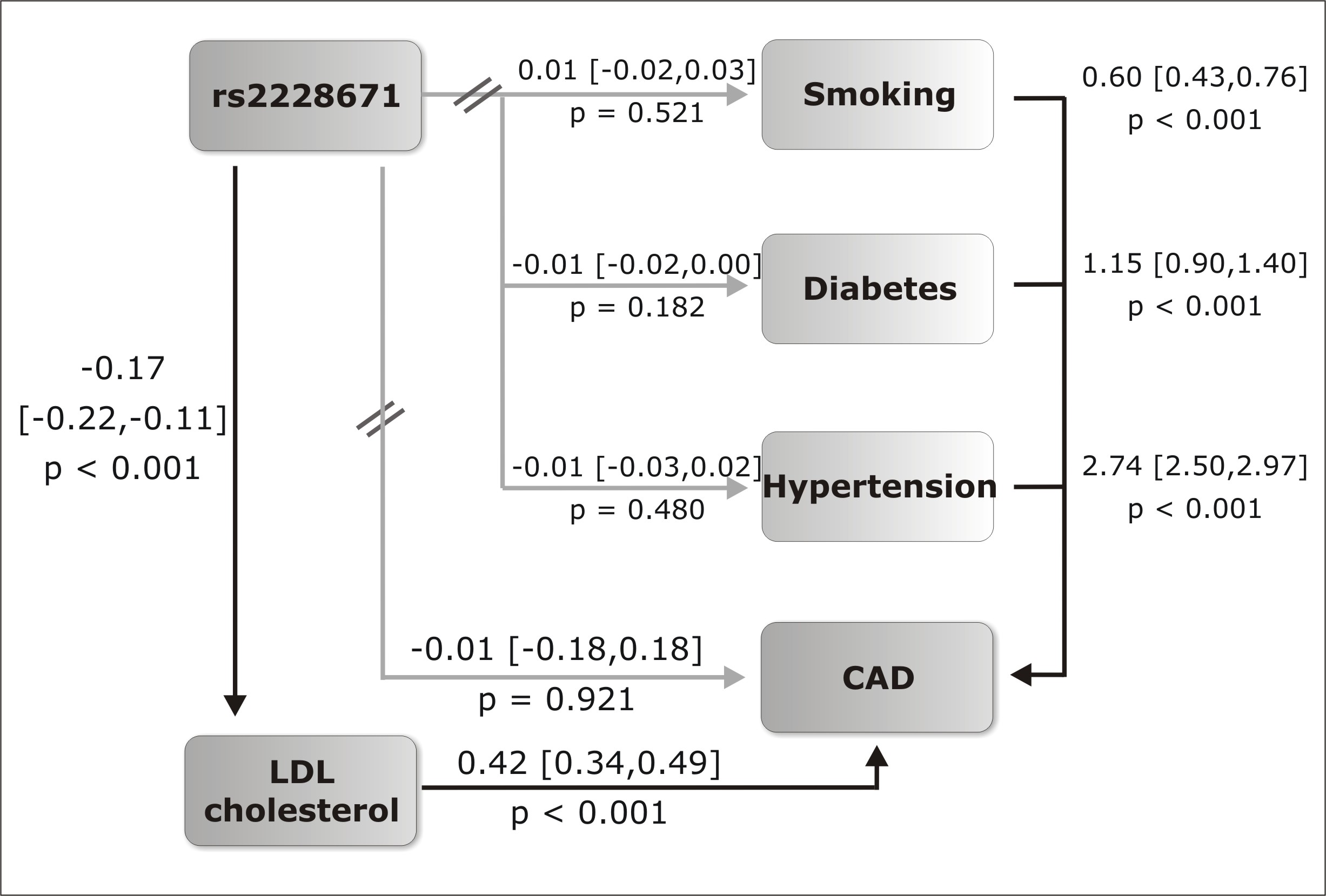


**Fig S1b**


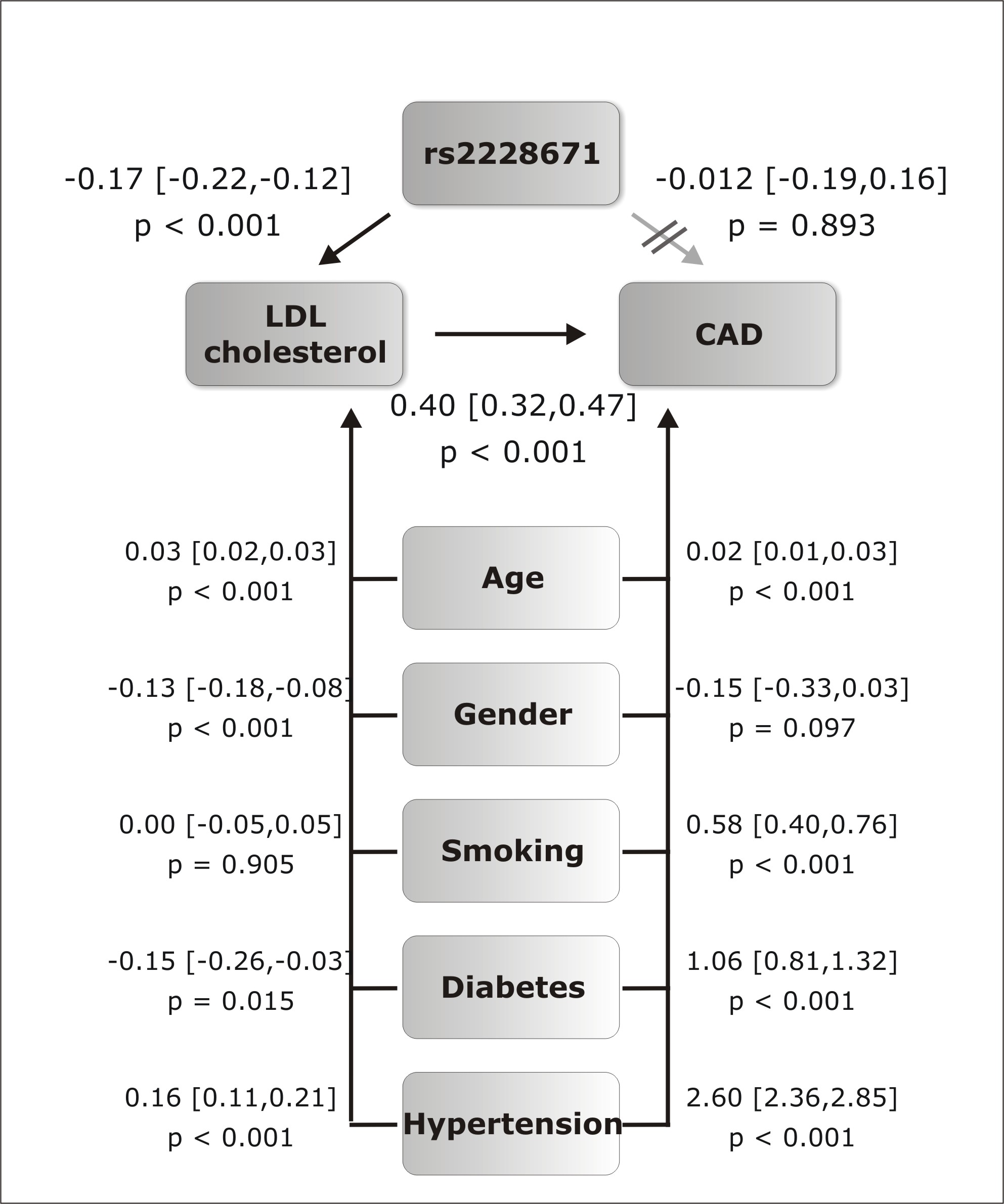


**Figure S1a and S1b:** Results of Mendelian randomisation study including known confounders.

Exploration of the causal relationship between LDL-C associated with rs2228671 and CAD (Mendelian Randomisation) including age, gender, smoking status, diabetes, and hypertension as known confounders. As in the simpler model (Fig. 5), the structural equation model shows that carriage of the T allele at rs2228671 leads to lower LDL-C levels, and higher LDL-C levels lead to an increased risk of CAD, whereas there is no additional direct path from rs2228671 to CAD risk, indicating that the functional pathway between the genetic variant at the LDLR gene locus and risk of CAD is through changes in LDL-C.

Part A: Higher age, male gender, hypertension and no diabetes lead to higher LDL-C levels, whereas higher age, smoking, hypertension and diabetes increase CAD risk.

Part B: Smoking, hypertension and diabetes increase CAD risk, but these confounders are not associated with rs2228671.
